# Improved production of fatty acid ethyl esters in *Saccharomyces cerevisiae* through up-regulation of the ethanol degradation pathway and expression of the heterologous phosphoketolase pathway

**DOI:** 10.1186/1475-2859-13-39

**Published:** 2014-03-12

**Authors:** Bouke Wim de Jong, Shuobo Shi, Verena Siewers, Jens Nielsen

**Affiliations:** 1 Department of Chemical and Biological Engineering, Chalmers University of Technology, Kemivägen 10, Göteborg SE-412 96, Sweden; 2 Institute of Chemical and Engineering Sciences, A-Star, 31, Biopolis Way, #01-01 Nanos, Singapore 138669, Singapore

**Keywords:** *Saccharomyces cerevisiae*, Fatty acid ethyl ester (FAEE), Biodiesel, Metabolic engineering

## Abstract

**Background:**

Due to an increasing demand of transportation fuels, a lower availability of cheap crude oil and a lack of sustainability of fossil fuels, a gradual shift from petroleum based fuels towards alternative and renewable fuel resources will be required in the near future. Fatty acid ethyl esters (FAEEs) have properties similar to current crude diesel and could therefore form an important contribution to the development of sustainable transportation fuels in future. It is important to develop novel cell factories for efficient production of FAEEs and their precursors.

**Results:**

Here, a *Saccharomyces cerevisiae* cell factory expressing a heterologous wax ester synthase (*ws2*) from *Marinobacter hydrocarbonoclasticus* was used to produce FAEEs from ethanol and acyl-coenzyme A (acyl-CoA). The production of acyl-CoA requires large amounts of NADPH and acetyl-CoA. Therefore, two metabolic engineering strategies for improved provision of NADPH and acetyl-CoA were evaluated. First, the ethanol degradation pathway was employed to re-channel carbon flow towards the synthesis of acetyl-CoA. Therefore, *ADH2* and *ALD6* encoding, respectively, alcohol dehydrogenase and acetaldehyde dehydrogenase were overexpressed together with the heterologous gene *acs*_*SE*_^*L641P*^ encoding acetyl-CoA synthetase. The co-overexpression of *ADH2*, *ALD6* and *acs*_*SE*_^*L641P*^ with *ws2* resulted in 408 ± 270 μg FAEE gCDW^−1^, a 3-fold improvement. Secondly, for the expression of the PHK pathway two genes, *xpkA* and *ack*, both descending from *Aspergillus nidulans*, were co-expressed together with *ws2* to catalyze, respectively, the conversion of xylulose-5-phosphate to acetyl phosphate and glyceraldehyde-3-phosphate and acetyl phosphate to acetate. Alternatively, *ack* was substituted with *pta* from *Bacillus subtilis*, encoding phosphotransacetylase for the conversion of acetyl phosphate to acetyl-CoA. Both PHK pathways were additionally expressed in a strain with multiple chromosomally integrated *ws2* gene, which resulted in respectively 5100 ± 509 and 4670 ± 379 μg FAEE gCDW^−1^, an up to 1.7-fold improvement.

**Conclusion:**

Two different strategies for engineering of the central carbon metabolism for efficient provision of acetyl-CoA and NADPH required for fatty acid biosynthesis and hence FAEE production were evaluated and it was found that both the ethanol degradation pathway as well as the phosphoketolase pathway improve the yield of FAEEs.

## Background

There is much interest in developing novel cell factories for production of advanced biofuels that can be used as diesel and bunker oil [1], and here we describe production of fatty acid ethyl esters (FAEEs) by yeast. The principle of FAEE production has been demonstrated in *Escherichia coli* as well as in *Saccharomyces cerevisiae* where FAEEs were formed by a transesterification reaction between ethanol and fatty acyl-CoA, which was catalyzed by a heterologous wax ester synthase/acyl-CoA:diacylglycerol acyltransferase (WS/DGAT) [2,3]. The enzyme activities of several WS/DGATs have been analyzed [4]. Five different wax ester synthase genes from different origins were expressed in *S. cerevisiae* and a strain expressing a wax ester synthase (*ws2*) from *Marinobacter hydrocarbonoclasticus* DSM 8798 showed the highest production of FAEEs [4,5].

While oleaginous organisms such as *Botryococcus braunii, Gordonia sp., Humicola lanuginose* or *Lipomyces starkeyi* were reported to accumulate lipids up to 65–75% of their cell dry weight, the yeast *S. cerevisiae* is a natural ethanol producer and not an oleaginous organism. There is therefore a demand for engineering strategies targeting the synthesis of acyl-CoA, one of the precursors for FAEE production [6-10].

The precursor for fatty acid biosynthesis is acetyl coenzyme A (acetyl-CoA), which plays a central role in several cellular pathways and compartments, and is involved in regulatory mechanisms [11,12]. In *S. cerevisiae,* it is produced in the cytosol, the mitochondria and the peroxisomes, but is not able to cross the organelle membranes. Acetyl-CoA also functions as a gateway for metabolic routes to many other biotechnologically valuable compounds, the pathways of which are mostly targeted to the cytosol and therefore there consists an interest to increase the production of acetyl-CoA in the cytosol [12,13].

**Figure 1:**
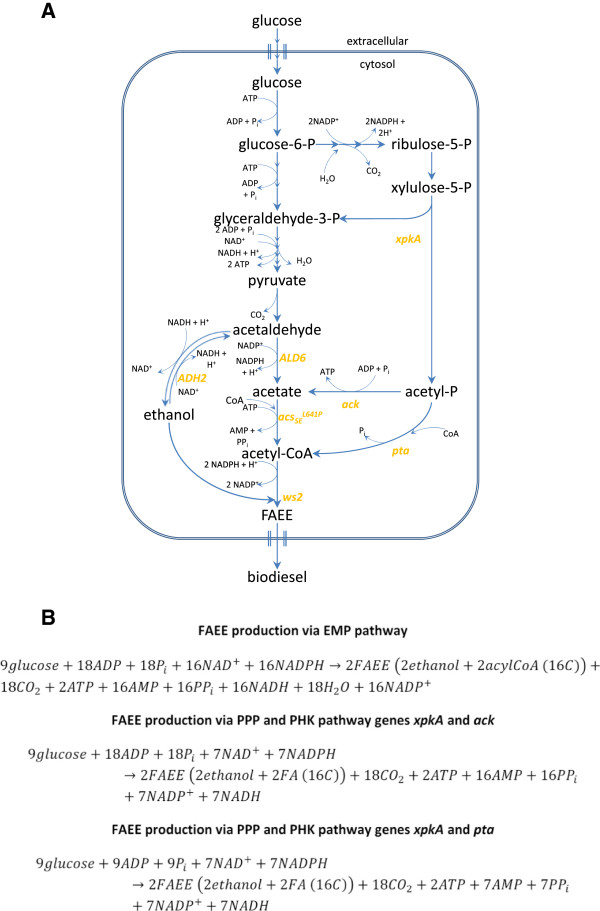
**Overview of metabolic engineering strategies. A**. Schematic metabolic pathways describing the (over-)expression of the ethanol degradation pathway and the phosphoketolase pathway. The genes of interest are: *xpkA* (xylulose-5-phosphate phosphoketolase), *ack* (acetate kinase), *pta* (phosphotransacetylase), *ADH2* (alcohol dehydrogenase 2), *ALD6* (acetaldehyde dehydrogenase), *acs*_*SE*_^*L641P*^ (acetyl-CoA synthetase) and *ws2* (wax ester synthase). **B**. Stoichiometric equations for the conversion of glucose to the product of interest, FAEE, via either the EMP pathway or the combination of PP and PHK pathway. For convenience it was assumed that the acyl-CoA chain was unsaturated and 16 carbons long. Abbreviations: ADP (adenosine diphosphate), NAD^+^ (nicotinamide adenine dinucleotide), NADPH (nicotinamide adenine dinucleotide phosphate hydrogenase), ATP (adenosine triphosphate), NADH (nicotinamide adenine dinucleotide hydrogenase), NADP^+^ (nicotinamide adenine dinucleotide phosphate).

During growth of *S. cerevisiae* on high concentrations of glucose, most glycolytic carbon is directed towards the production of ethanol from acetaldehyde which is known as the Crabtree effect [14,15]. To reduce the amount of ethanol and increase the amount of cytosolic acetyl-CoA, a metabolic engineering strategy described by Chen et al. [13] was applied. Alcohol dehydrogenase 2 (Adh2) catalyzes the conversion of ethanol to acetaldehyde in *S. cerevisiae.* For the formation of cytosolic acetyl-CoA, acetaldehyde is converted to acetate, which is catalyzed by acetaldehyde dehydrogenase encoded by endogenous *ALD1 - ALD7* and subsequently to acetyl-CoA catalyzed by acetyl-CoA synthetase (encoded by *ACS1* and *ACS2*) [12,16]. In this approach, endogenous *ALD6* and a heterologous *ACS* variant from *Salmonella enterica* encoded by *acs*_*SE*_^*L641P*^ were overexpressed. The ACS variant *acs*_*SE*_^*L641P*^ carries an amino acid substitution that prevents the enzyme from being inactivated by acetylation [17,18]. The metabolic pathway is shown in Figure 1A.

For the synthesis of acyl-CoA, acetyl-CoA forms the primer which is extended by addition of C2 units derived from malonyl-CoA. For each new addition of a C2 unit derived from malonyl-CoA the chain grows with two carbons until an average length of 16 carbons is reached. However, each reaction cycle includes two reduction steps, each requiring the redox co-factor NADPH [19]. Therefore, another metabolic pathway leading towards the synthesis of acetyl-CoA was investigated.

The phosphoketolase (PHK) pathway was described previously as potential alternative carbon route for different industrially relevant metabolites due to the increase of acetyl-CoA production with NADPH as co-product [20-22]. The carbon flow leading towards the PHK pathway starts with the conversion of glucose to glucose-6-phosphate in the Embden-Meyerhof-Parnas (EMP) pathway followed by conversion of glucose-6-phosphate in three reactions into ribulose-5-phosphate in the oxidative part of the pentose phosphate pathway (PPP). Byproducts during these reactions are two moles of NADPH and one mole of CO_2_ per mole of glucose. The next metabolite in the pathway, xylulose-5-phosphate, represents the precursor of the PHK pathway as it can be converted to acetyl-phosphate and glyceraldehyde-3-phosphate. This reaction is catalyzed by xylulose-5-phosphate phosphoketolase encoded by *xpkA* in *Aspergillus nidulans*. During the second step of the PHK pathway, acetyl phosphate can either directly be converted into acetyl-CoA or indirectly with acetate as intermediate. Direct conversion of acetyl phosphate into acetyl-CoA in *Bacillus subtilis* is catalyzed by phosphotransacetylase encoded by the gene *pta*[23]. The formation of acetate and ATP from acetyl phosphate is performed by acetate kinase, encoded by the gene *ack* in *A. nidulans*[24,25]. The metabolic pathway is shown in Figure 1A.

The PHK pathway was first reported in heterofermentative and facultative homofermentative lactic acid bacteria, in bifidobacteria and sporadic in other microorganism, like xylose fermenting yeasts [26]. It has been demonstrated that *S. cerevisiae* has the ability to functionally express phosphotransacetylase from *B. subtilis* as well as phosphoketolase and acetate kinase from *A. nidulans*[20,27]. The fact that more NADPH is formed when glucose is metabolized via the PPP instead of the EMP pathway makes the combination of PPP and PHK pathway an interesting alternative for FAEE production. The demand of NADPH for lipid synthesis might create the driving force for carbon to flow through the PHK pathway. The reaction equations in Figure 1B show that glucose catabolism through the PHK pathway would reduce the net NADPH demand for FAEE production.

Here, the research goal was the improvement of a *S. cerevisiae* FAEE cell factory. In more detail, *ws2* from *M. hydrocarbonoclasticus*[4] was expressed in combination with the two different pathways mentioned above, i.e. the ‘ethanol degradation’ pathway responsible for supplying precursor acetyl-CoA and the heterologous phosphoketolase pathway supplying both acetyl-CoA and redox co-factor NADPH. The results of expressing the two pathways were compared in terms of physiological properties and FAEE production of the recombinant strains.

## Materials and methods

### Strains

All plasmid constructions were performed with *E. coli* strain DH5α [28]. Yeast strain *S. cerevisiae* CEN.PK 113-11C (*MAT***a***MAL2*-*8*^*c*^*SUC2 ura3-52 his3-*Δ*1*) was kindly provided by P. Kötter, University of Frankfurt, Germany. *S. cerevisiae* CB2I20 is a derivative of CEN.PK 113-5D (*MAT***a***MAL2*-*8*^*c*^*SUC2 ura3-52)* and contains multiple *ws2* chromosomal integrations. This strain has been used for stable expression of *ws2* and expression of genes encoding endogenous acyl-CoA binding protein (*ACB1*) and a bacterial NADP^+^-dependent glyceraldehyde-3-phosphate dehydrogenase (*gapN*) (unpublished).

### Media and growth conditions

*E. coli* cells were cultured at 37°C and 200 rpm in lysogeny broth (LB) [29] containing 80 mg l^−1^ ampicillin when needed. *S. cerevisiae* strains were cultured at 30°C and 150 rpm in synthetic dextrose (SD) medium containing 20 g l^−1^ glucose, 6.7 g l^−1^ yeast nitrogen base without amino acids (YNB-AA) (Formedium, Hunstanton, UK), and complete supplement mixture (0.750 g l^−1^; CSM, Formedium) lacking uracil and histidine.

### Plasmid construction

Plasmids containing a *HIS3* selection marker were derived from episomal 2-micron plasmids pIYC04 and pIYC08. The construction of the plasmids was described by Chen et al. [12,13]. The codon optimized *ws2* gene from *M. hydrocarbonoclasticus* (Menlo Park, CA, USA) [4] was amplified with forward primer CTTCAA*ACTAGT*AAAACAATGAAGAGATTAGGTACTCTAGACG and reverse primer CTTCTT*GAGCTC*TTACTTTCTAGTACGGGCACG attaching restriction sites *Spe*I and *Sac*I (marked *italic*) to the gene. This fragment was cloned into a multi-cloning site on pIYC04 resulting in a plasmid containing the *ws2* gene under control of the *TEF1* promoter and the *ADH1* terminator. This plasmid was verified by sequencing (Eurofins MWG Operon, Ebersberg, Germany) and named pBDJ01. pBDJ02 was constructed from plasmids pIYC08 and pBDJ01, which were both restricted with *Sac*I and *Spe*I. The insert from pBDJ01 containing the *ws2* gene was cloned into the pIYC08 backbone containing *ADH2*, *ALD6* and *acs*_*SE*_^*L641P*^. pBDJ02 finally contained four genes: *ws2*, *ALD6*, *ADH2* and *acs*_*SE*_^*L641P*^. The construction of an additional plasmid (pIYC09) containing genes *ALD6*, *ADH2* and *acs*_*SE*_^*L641P*^ was described by Chen et al. [30]. Plasmid pSP-GM2 [31,32] was used as a backbone for the construction of plasmids that contained the *URA3* selection marker. The *ws2* gene was restricted with *Not*I/*Sac*I from pBdJ02 and then ligated into the *Not*I/*Sac*I sites of vector pSP-GM2 to construct pSP-B2N. The construction of plasmid pMPa containing genes *xpkA* and *ack* was described previously [20]. Plasmid pMPp was constructed accordingly with the difference that it contained *pta* instead of *ack*. All plasmids were confirmed by sequencing (Eurofins). A list of all plasmids is shown in Table 1.

**Table 1:** List of plasmids used in this study

**Plasmid name**	**Genes**	**Marker gene**	**Source**
**pIYC04**	*---*	*HIS3*	[12]
**pBdJ01**	*ws2*	*HIS3*	This study
**pICY09**	*ALD6, ADH2, acs*_*SE*_^*L641P*^	*HIS3*	[13]
**pBdJ02**	*ws2, ALD6, ADH2, acs*_*SE*_^*L641P*^	*HIS3*	This study
**pSP-GM2**	*---*	*URA3*	[31]
**pSPB2N**	*ws2*	*URA3*	Shi et al., submitted
**pMPa**	*xpKA, ack*	*URA3*	[21]
**pMPp**	*xpKA, pta*	*URA3*	This study

### Strain construction and transformation

The different yeast strains constructed are listed in Table 2. Transformations were performed by following the lithium acetate/single-stranded carrier DNA/polyethylene glycol method [33].

**Table 2:** Strains used in this study

**Name**	**Genetic background**	***HIS3*** **based plasmid**	***URA3*** **based plasmid**
**Reference strain**			
**BdJref**	CEN.PK 113-11C	pIYC04 (−−*-*)	pSP-GM2 (−−*-*)
**Phosphoketolase strains**			
**BdJ01**	CEN.PK 113-11C	pBdJ01 (*ws2*)	pSP-GM2 (−−-)
**BdJ02**	CEN.PK 113-11C	pBdJ01 (*ws2*)	pMPa (*xpkA, acK*)
**BdJ03**	CEN.PK 113-11C	pBdJ01 (*ws2*)	pMPp (*xpkA, pta*)
**BdJ04**	CB2I20	*---*	pSP-GM2 (−−-)
**BdJ05**	CB2I20	*---*	pMPa (*xpkA, acK*)
**BdJ06**	CB2I20	*---*	pMPp (*xpkA, pta*)
**Ethanol degradation strains**			
**BdJ07**	CEN.PK 113-11C	pIYC04 (−−*-*)	pSPB2N (*ws2*)
**BdJ08**	CEN.PK 113-11C	pBdJ02 (*ws2, ALD6, ADH2, acs*_*SE*_^*L641P*^)	pSP-GM2 (−−-)
**BdJ09**	CEN.PK 113-11C	pIYC09 (*ALD6, ADH2, acs*_*SE*_^*L641P*^)	pSPB2N (*ws2*)

### Culturing

The different *S. cerevisiae* strains were stored in 15% glycerol at −80°C before they were pre-cultured in 5 ml selective SD medium at 30°C and 200 rpm. Thereafter, the strains were grown in 500 ml shake flasks containing 50 ml selective SD-medium starting with an optical density (OD_600_) of 0.01. Cultures were grown for up to 100 hours at 30°C and 150 rpm.

### Analysis of physiological parameters

While culturing, samples were taken regularly (every 3–4 h). The cell growth was measured with a Genesys 20 spectrophotometer (Thermo Fisher Scientific Inc., Waltham, MA, USA) by determining the optical density at 600 nm. The concentration of remaining substrate and formation of byproducts (ethanol and glycerol) was analyzed using filtered samples (0.2 μm nylon membrane) by high performance liquid chromatography (HPLC; Dionex Ultimate 3000 HPLC system Dionex Softron GmbH, Germering, Germany) equipped with an Aminex HPX-87H column (Bio-Rad, Hercules, CA, USA) at 65°C and fed with a mobile phase of 5 mM H_2_SO_4_ at a flow rate of 0.6 ml min^−1^. Glucose, glycerol and ethanol were measured with a Shodex RI-101 refractive index detector (Showa Denko, Tokyo, Japan).

### Sample preparation for quantification of FAEEs

After 100 hours of culturing the remaining biomass was centrifuged and washed with MQ water thrice before being freeze-dried (Christ Alpha 2–4 LSC, Shropshire, UK). Further sample treatment was performed with a known amount of cell dry weight (CDW) and 20 μg of heptadecanoic acid ethyl ester (17:0) as internal standard (IS). This sample was mixed with 7 ml CHCl_3_ : MeOH (2:1, v/v) in an extraction tube (Pyrex borosilicate glass 16 × 100 mm) and flushed with nitrogen gas until all air was removed before total lipids were extracted from the sample by a microwave-assisted method described previously [34]. Next, 1.7 ml NaCl (0.73%, w/v) was added to the sample and vortexed vigorously. Centrifugation at 3000 rpm for 5 min resulted in a phase separation. The organic (lower) phase was transferred into a clean tube for vacuum evaporation using a miVac concentrator (Genevac, Ipswich, UK). The sample was re-suspended in 50 μl CHCl_3_ : MeOH (2:1, v/v), loaded onto a thin layer chromatographic (TLC) Silica gel 60 F254plate (Merck, Darmstadt, Germany) and separated using a mobile phase of heptane, 2-propanol and acetic acid with a ratio of 95:5:1 (v/v/v) [4]. Free fatty acids, triacylglycerols (TAGs), sterol esters and FAEEs were identified using a standard solution containing heptadecanoic acid, glyceryl triheptadecanoate, cholesteryl palmitate and heptadecanoic acid ethyl ester (Sigma-Aldrich, St. Louis, MO, USA). Visualization was performed under ultraviolet radiation after spraying with 0.05% 2,7-dichlorofluorescein in ethanol. The spot of the TLC plate corresponding to FAEEs was scraped off with a razor blade and collected in a tube containing a mix of 3 ml hexane, 2 ml methanol and 2 ml MQ water. The tube was vigorously vortexed, and after centrifugation at 3000 rpm for 5 min, the upper layer was transferred to a clean tube. This solution, containing the FAEEs, was dried by vacuum evaporation using a miVac concentrator (Genevac, Ipswich, UK) before the sample was finally dissolved in 1 ml hexane.

### GC-MS analysis

The equipment for separating and quantifying FAEEs included a Focus GC ICQ single quadruple GC-MS from Thermo Fisher Scientific with a Zebron (ZB-WAX) GC column with 30 m × 0.25 mm internal diameter and 0.25 μm film thickness (Phenomenex, Macclesfield, UK). The precise GC-MS conditions, compound identification and quantification conditions have previously been described [4].

## Results

### FAEE production in strains expressing the ethanol degradation pathway

The production of FAEEs requires a large amount of acetyl-CoA and therefore it was a straight forward idea to metabolically engineer the native pathway in *S. cerevisiae* to increase the flux towards cytosolic acetyl-CoA synthesis. Ethanol, the second metabolite for synthesis of FAEEs, forms the major byproduct during FAEE production in *S. cerevisiae*. To re-channel the carbon flux towards the synthesis of acetyl-CoA, Adh2 and Ald6, which respectively catalyze the conversion of ethanol to acetaldehyde and acetaldehyde to acetate, were overexpressed together with the heterologous *acs*_*SE*_^*L641P*^, which encodes a de-regulated acetyl-CoA synthetase that catalyzes the conversion of acetate to acetyl-CoA. Details about the metabolic pathway and the strain construction are shown in Figure 1 and Table 2, respectively.

**Figure 2:**
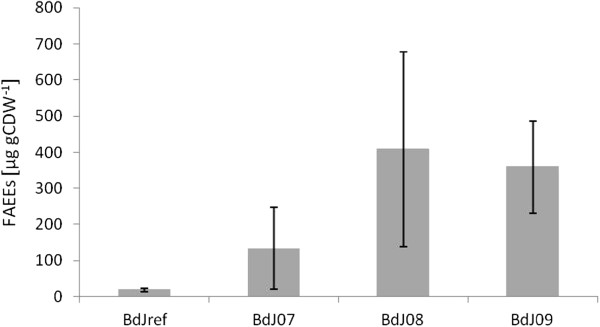
**FAEE yield (μg gCDW**^**−1**^**) of investigated *S. cerevisiae *strains expressing the ethanol degradation pathway.** Biological triplicates of the strains were investigated. The experiment was performed twice and standard deviations are indicated.

In Figure 2, it can be observed that the introduction of a wax ester synthase resulted in a FAEE yield of 133 ± 113 μg gCDW^−1^ (strain BdJ07). Up-regulation of *ALD6*, *ADH2* and *acs*_*SE*_^*L641P*^, expressed from the same plasmid, led to a yield of 408 ± 270 μg gCDW^−1^ (strain BdJ08), a 3-fold improvement compared to strain BdJ07, which however lacks statistical significance (p-value: 0.08). The strain showed a large variation of the FAEE yield in the different clones measured, which might be caused by variations in copy number of the relatively large plasmid. Therefore, a similar strain was constructed, reducing the plasmid size by expressing the genes on two different plasmids. This strain, BdJ09, in which *ws2* and the three genes *ALD6*, *ADH2* and *acs*_*SE*_^*L641P*^ were expressed from separate plasmids resulted in a FAEE yield of 359 ± 128 μg gCDW^−1^. This modified strain showed a 2.7 fold improvement compared to strain BdJ07, which only expressed *ws2* (p-value: 0.03), and also showed a lower clonal variation than strain BdJ08.

### FAEE production in strains expressing the PHK pathway

**Figure 3:**
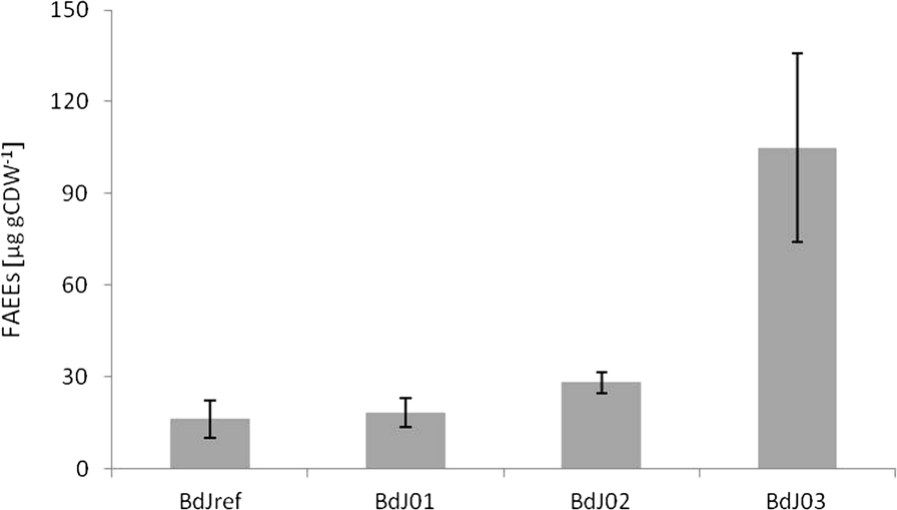
**FAEE yield (μg gCDW**^**−1**^**) of investigated *S. cerevisiae *strains expressing a heterologous PHK pathway.** Biological triplicates of the strains were investigated. The standard deviations are indicated.

Introduction of the PHK pathway has the potential to increase the yield of FAEEs in *S. cerevisiae* due to its ability to generate two moles of NADPH per mole of glucose, which would provide additional NADPH for acyl-CoA synthesis. Therefore, two genes, *xpkA* and *ack*, both descending from *A. nidulans*, were expressed to catalyze, respectively, the conversion of xylulose-5-phosphate to acetyl phosphate and glyceraldehyde-3-phosphate and acetyl phosphate to acetate with the gain of one ATP. A second strain was constructed, in which the gene *pta* from *B. subtilis* replaced *ack*. Pta converts acetyl phosphate directly to acetyl-CoA. Both strains were compared with strain BdJ01 containing solely the *ws2* gene and to a reference strain (BdJref) containing the empty reference plasmids. Figure 3 shows the quantification of total FAEEs of the described *S. cerevisiae* strains. The expression of *xpkA*, *ack* and *ws2* (strain BdJ02) resulted in a FAEE yield of 28 ± 3.5 μg gCDW^−1^ which is 1.5 times higher than for the strain only expressing *ws2* (BdJ01). However, the difference between the two strains showed insufficient statistical significance (p-value: 0.10). The expression of *xpkA*, *pta* and *ws2* (BdJ03) led to a FAEE yield of 105 ± 30 μg gCDW^−1^ which was 5.7 fold higher than the strain with solely *ws2* expression (p-value: 0.03) and a 3.7 times higher FAEE yield than the strain expressing *xpkA*, *ack* and *ws2 (p-value: 0.05)*.

**Figure 4:**
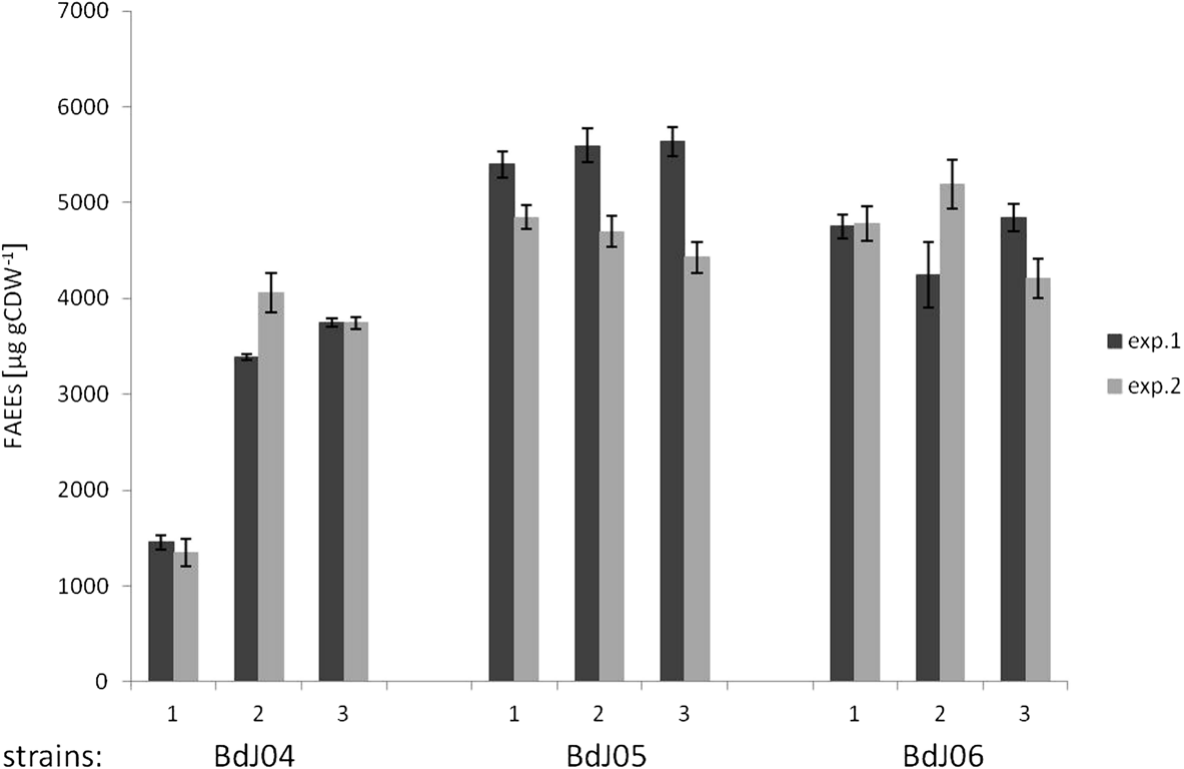
**FAEE yield (μg gCDW**^**−1**^**) of investigated *S. cerevisiae *strains containing multiple *ws2 *chromosomal integrations and expressing a heterologous PHK pathway.** Triplicate clones of each strain were investigated (described by 1, 2 and 3). The experiment was performed twice (light and dark columns) and standard deviations are indicated.

Due to the relatively low yield of the strains described above and because of presumed fluctuating plasmid stability during repetitive experiments, the PHK pathway was also expressed in strain CB2I20, a strain with multiple chromosomal *ws2* integrations (Shi et al., submitted). These strains are listed in Table 2. It was also hypothesized that multiple expression of the *ws2* gene might contribute to a stronger carbon-pull through the PHK pathway. Expression of *xpkA* and *pta* in strain CB2I20 (BdJ06) resulted in 4670 ± 379 μg gCDW^−1^ (p-value: 0.02), a 1.6 fold higher yield of FAEEs compared to the reference strain CB2I20 (BdJ04), whereas the expression of *xpkA* and *ack* in CB2I20 (BdJ05) improved the final yield of FAEEs to 5100 ± 509 μg gCDW^−1^ (p-value: 0.01), which is a 1.7 times improvement compared to BdJ04 (Figure 4). It could also be observed that the production levels of FAEEs in strains BdJ04-BdJ06 were stable and reproducible.

### Physiological data

**Figure 5:**
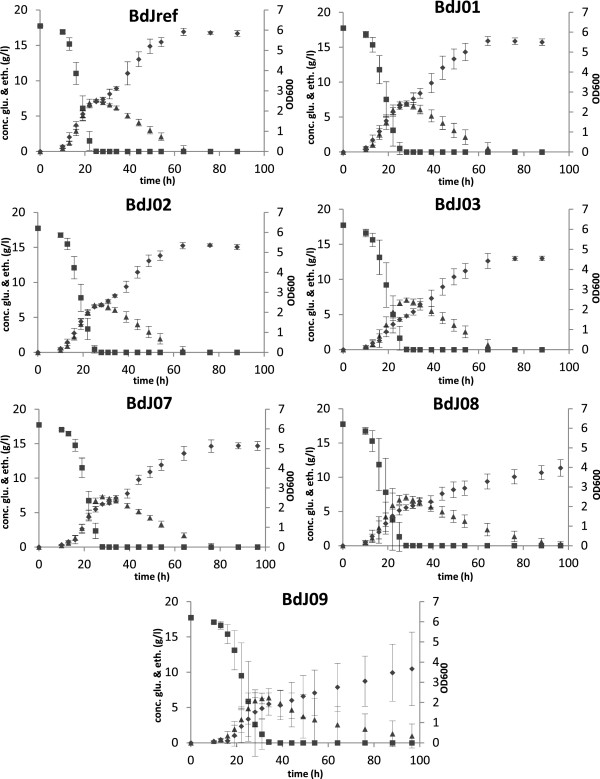
**Cell growth and substrate consumption of recombinant strains during shake flask cultivation.** The strains BdJref and BdJ01-BdJ06 were analyzed in biological triplicates. The glucose concentration (g l^−1^) is indicated by squares, the ethanol concentration (g l^−1^) is indicated by triangles and cell growth (OD 600) is shown by diamonds.

Wild type *S. cerevisiae* is a fast growing and robust microorganism. However, the genetic modifications leading to improved FAEE production could have an impact on its physiology. Cell growth, glucose consumption as well as ethanol formation and consumption were therefore monitored during shake flask cultivations (Table 3 and Figure 5). The final biomass of the engineered strains was reduced compared to the reference strain (BdJref) and also the maximum specific growth rate (μ_max_) for both PHK pathway expressing strains and ethanol degradation pathway engineered strains showed a reduction of up to 40% (BdJ08). The maximal glucose consumption rate (q_smax_) was only slightly reduced for the engineered strains if compared to the reference strain. The most noticeable physiological change between the strains expressing the PHK pathway and the strains engineered for increased ethanol degradation was a slower maximal consumption rate of ethanol (q_ethmax_) for the latter (strains BdJ07-BdJ09). Especially the strains overexpressing genes *ADH2*, *ALD6*, *acs*_*SE*_^*L641P*^ and *ws2* showed a reduced ethanol consumption rate of, respectively, 45% (BdJ08) and 70% (BdJ09) compared to the reference strain.

**Table 3:** Physiological characteristics of modified strains

**Strain**	**(Over)expressed genes( *HIS3 *** **plasmid) &( *URA3 *** **plasmid)**	**Final FAEE yield (μg gCDW**^**−1**^**)**	**Maximal specific growth rate (μ**_**max**_**)**	**Final biomass (OD 600)**	**Glucose consumption rate (q**_**smax**_**)**	**Ethanol consumption rate (q**_**ethmax**_**)**
**BdJref**	--- & ---	16.3 ± 3.5	0.190 ± 0.010	5.84 ± 0.14	−1.534 ± 0.054	−0.204 ± 0.015
**Phosphoketolase pathway**						
**BdJ01**	*ws2 & ---*	18 ± 4.8	0.159 ± 0.006	5.51 ± 0.16	−1.448 ± 0.057	−0.200 ± 0.021
**BdJ02**	*ws2 & xpkA, ack*	28.0 ± 3.5	0.168 ± 0.009	5.27 ± 0.13	−1.357 ± 0.084	−0.208 ± 0.016
**BdJ03**	*ws2 & xpkA, pta*	105 ± 30	0.136 ± 0.025	4.55 ± 0.11	−1.357 ± 0.078	−0.197 ± 0.014
**Ethanol degradation pathway**						
**BdJ07**	*--- & ws2*	133 ± 113	0.192 ± 0.003	5.14 ± 0.22	−1.403 ± 0.035	−0.174 ± 0.003
**BdJ08**	*ws2, ALD6, ADH2, acs*_*SE*_^*L641P*^ &*---*	408 ± 270	0.115 ± 0.026	3.97 ± 0.42	−1.265 ± 0.365	−0.113 ± 0.002
**BdJ09**	*ALD6, ADH2, acs*_*SE*_^*L641P*^ &*ws2*	359 ± 128	0.136 ± 0.035	3.67 ± 1.82	−1.208 ± 0.495	−0.061 ± 0.023

## Discussion

The production of FAEEs in yeast *S. cerevisiae* could form an important contribution to the development of sustainable diesel transportation fuels in the future. However, to turn *S. cerevisiae* into an efficient FAEE producer instead of an ethanol producer, its metabolism requires further engineering. FAEEs are synthesized from acyl-CoA and ethanol and the formation of acyl-CoA in turn is dependent on precursors acetyl-CoA and there from derived malonyl-CoA. Acyl-CoA formation also requires a large amount of co-factor NADPH. In this study, two different metabolic engineering strategies were applied to increase the supply of carbon and co-factor NADPH for production of the FAEE precursor acyl-CoA.

The two main metabolic reactions in *S. cerevisiae* to produce NADPH during growth on glucose are the conversion of acetaldehyde to acetate by acetaldehyde dehydrogenase and the first steps in the pentose phosphate pathway (catalyzed by glucose-6-phosphate dehydrogenase and 6-phosphogluconate dehydrogenase). Grabowska et al. demonstrated this by constructing a *S. cerevisiae* mutant with deletions in *ZWF1* (encoding glucose-6-phosphate dehydrogenase) and *ALD6* (encoding cytosolic aldehyde dehydrogenase), which was not viable on glucose [35]. Later, an additional source of NADPH was identified during growth on lactate, namely cytosolic isocitrate dehydrogenase (Idp2) [36].

In this study, it was found that expression of the phosphoketolase pathway in *S. cerevisiae* could re-channel carbon flux through the oxidative part of the PPP towards the precursors acetyl-CoA and malonyl-CoA for synthesis of acyl-CoA. As a consequence, two molecules of the NADPH are being produced for each sugar molecule passing through the PPP. As was demonstrated by Papini et al. the expression of genes *xpkA* and *ack* showed a functional carbon flux through the PHK pathway and as shown in this study the expression of genes *xpkA*, *ack* and *ws2* resulted in a yield of FAEEs 50% higher than in a strain not expressing the PHK pathway [20]. It was also shown that the expression of *xpkA*, *ack* resulted in an improved polyhydroxybutyrate (PHB) producing strain [22]. Papini et al. could not demonstrate the functionality of the PHK pathway using phosphotransacetylase gene *pta* (instead of *ack*), which would result in a direct conversion of acetyl phosphate to acetyl-CoA. However, in their study they did not insert a pull of acetyl-CoA for a specific product, whereas our result clearly shows that expression of this enzyme combination results in improved FAEE production (minimal 60% improvement), which strongly indicates that the enzymes are active. We therefore conclude that the expression of *ack* from *A. nidulans* or *pta* from *B. subtillis* combined with expression of *xpkA* from *A. nidulans* and *ws2* from *M. hydrocarbonoclasticus* represents a successful strategy for increasing FAEE production by *S. cerevisiae*.

The second strategy, the ethanol degradation pathway, was based on the native acetyl-CoA supply in the cytosol of *S. cerevisiae*. Chen et al. investigated the physiological effect of some major enzymes relevant for acetyl-CoA metabolism on different carbon sources in *S. cerevisiae*[12]. Besides being regulated at the transcriptional and post-translational level, yeast acetyl-CoA synthetases Acs1 and Acs2 also show differential subcellular localization [12,37]. It was shown that the posttranslationally regulated acetyl-CoA synthetase in *S. enterica* is prevented from acetylation by a point mutation substituting leucine for proline at position 641 of the enzyme and maintaining it in its active state [18]. This enzyme variant encoded by *acs*_*SE*_^*L641P*^ was previously overexpressed together with *ALD6*, mainly responsible for the metabolic reaction to form acetate from acetaldehyde, in *S. cerevisiae* for high-level production of amorphadiene [17]. This successful strategy had been improved by additional overexpression of *ADH2* and *ERG10* for the production of α-santalene which resulted in a 1.75 times higher production than the reference strain (without overexpression of the four genes) and a 25% increase in titer compared to the strategy introduced by Shiba et al. [17]. The pull-push strategy, pushing the carbon flow down to acetyl-CoA by overexpression of *ADH2*, ALD6 and *acs*_*SE*_^*L641P*^ and pulling it towards the product by over expression of *ERG10* was also applied in the production of polyhydroxybutyrate (PHB) in *S. cerevisiae*[38]. A 16-fold improvement of PHB production was detected compared to the reference strain. In this study, the wax ester synthase was used to pull the carbon flow towards production of FAEEs. Comparable to the previously performed studies described, we here found the improvement of product formation to be 3-fold compared with the reference strain.

It remains important to consider the different platforms for gene expression in *S. cerevisiae*. In general, the expression can occur from plasmids or genes can be integrated into the chromosomes. Here both engineered pathways were expressed on 2-micron plasmids resulting in a high copy numbers and therefore high expression. However, expression of several genes on 2-micron plasmids results in large plasmids (pBdJ02 > 15 kb). Plasmid size as well as promoter strengths influences the stability of the plasmid and therefore the gene expression when propagated in *S. cerevisiae*[39-41]. We indeed saw a high clonal variation in some of our plasmid based strains. On the other hand, integration of genes into the chromosomes results in stable expression levels as was clearly demonstrated by Jensen et al. [42]. Strain CB2I20, which was used for expression of the PHK pathway, contained multiple (1–6) integrated copies of the *ws2* gene which resulted in stable production levels of FAEE with a 10 fold higher yield (Shi S, Valle-Rodríguez J, Siewers V, Nielsen J: Engineering of chromosomal wax ester synthase integrated *Saccharomyces cerevisiae* mutants for improved biosynthesis of fatty acid ethyl esters, submitted). The high yield in combination with stable expression levels reached by multiple integrated copies is especially valuable for industrial yeast strains.

Further, it seems unlikely that FAEE is toxic to *S. cerevisiae* at the concentrations produced here. There was no negative effect on cell growth when 1 g/l of myristic acid ethyl esters were added to the medium (data not shown). Therefore, the reduced ethanol consumption rates observed (45% in BdJ08 and 70% in BdJ09 during the ethanol degradation pathway might most likely be explained by the rapid conversion of the ethanol to cytosolic acetyl-CoA which hence reduces the amount of ethanol that can be oxidized in the mitochondria. In general, it might be a presumable assumption that the metabolic changes were caused by the overexpression of several genes.

Thus, in conclusion we found that both strategies for improving the supply of cytosolic acetyl-CoA and NADPH, i.e. overexpression of the heterologous PHK pathway or the overexpression of the ethanol degradation pathway, resulted in improvement in the production of FAEEs, and hence form the basis for engineering an efficient cell factory for production of this biodiesel component.

## Competing interest

The author’s declare that they have no competing interests.

## Authors’ contributions

BWDJ performed all the experiments and took part in designing the study, evaluating the results and writing the manuscript. SS, VS and JN took part in designing the study, evaluating the results and writing the manuscript. This manuscript has been approved by all the authors listed.
